# Comparison of Lower Extremity Alignment among Taekwondo Athletes of Various Subdisciplines

**DOI:** 10.3390/medicina60030493

**Published:** 2024-03-17

**Authors:** Mi-ock Han, Eun-wook Chang, Hyung-pil Jun

**Affiliations:** 1 Department of Physical Education, Dong-A University, Busan 49315, Republic of Korea; aldhr2927@gmail.com; 2 Department of Sports Science, Inha University, Incheon 22212, Republic of Korea; 3 Yonsei Institute of Sports Science and Exercise Medicine, Yonsei University, Seoul 03722, Republic of Korea

**Keywords:** taekwondo athletes, lower extremity alignment

## Abstract

*Background and Objectives:* Studies analyzing lower extremity alignment (LEA) LEA among taekwondo subdisciplines athletes are lacking. This study compared LEA in the dominant and nondominant legs intaekwondo athletes. *Materials and Methods:* Twelve measurements of LEA were analyzed for 157 athletes (63 sparring, 50 demonstration, and 44 poomsae athletes) registered with the Korea Taekwondo Association. LEA was measured in the standing, supine, and prone positions using alignment application, a goniometer, a bubble inclinometer, a height gage, and a palpation meter. *Results:* The analysis revealed that the dominant leg of poomsae athletes showed greater genu valgum alignment than that of sparring athletes (*p* < 0.01), whereas the dominant leg of sparring athletes showed greater rearfoot varum alignment than that of demonstration athletes (*p* < 0.01). Furthermore, the nondominant leg of poomsae athletes showed greater genu valgum alignment than that of sparring and demonstration athletes (*p* < 0.01), whereas the nondominant leg of sparring athletes showed greater rearfoot varum alignment than that of demonstration athletes (*p* < 0.01). In addition, demonstration athletes had better forefoot varus alignment than poomsae athletes (*p* < 0.01). *Conclusions:* This study revealed that LEA characteristics vary among taekwondo athletes according to their subdiscipline. The results of this study would help in designing training programs tailored to each subdiscipline that would best address their LEA characteristics and help to prevent injuries.

## 1. Introduction

The sport of taekwondo is divided into the subdisciplines of sparring, demonstration, and poomsae. Depending on the game rules and characteristics required for each specific event, athletes in these subdisciplines would exhibit differences in their kick angle, force, and physical factors [1,2,3]. In sparring, transitions between attack and defense techniques are constantly executed in anticipation of the opponent’s movements, with variations in striking distance and height. Demonstration is characterized by executing techniques and performances that demonstrate taekwondo movements within a short time. In addition, although movements in poomsae are more consistent than those in sparring and demonstration, the evaluation score varies depending on the kick height, which requires excessive flexibility and a wide range of motion (ROM) of the hip joints. Despite the differences in kicking methods required by the rules of the game and the distinct characteristics of each subdiscipline, they all involve similar kicking techniques and biomechanics, in that they require the execution of movements based on basic kicks [4].

To perform a stable kick, the attacking and nonattacking legs must interact simultaneously. Athletes attack with their dominant leg more frequently to perform successful techniques. Taekwondo techniques engage the foot to control the body position or distance to the target and require stability and postural control in the nondominant leg [5]. A previous study that investigated the plantar pressure of the dominant and nondominant legs found asymmetrical plantar pressure between the legs depending on the frequency of executing attacks [6]. In particular, subjects with high static balance errors of the nondominant leg showed lower forefoot plantar pressure than rearfoot plantar pressure in both feet [6].

The basic kick in taekwondo involves a rotational movement of each segment of the body, starting with the hip, followed by the knee, and then the ankle. The speed of the hip rotation varies depending on the height of the target [4,7]. Kinematic analysis has shown that the speed of the distal segment depends on the speed of the proximal segment and the interaction between each segment [8]. Kicking at high speeds has a positive effect on scoring and continuous skills, but it also increases soft tissue tension due to repeated momentary muscle contractions [9]. Increased tension in soft tissues can lead to musculoskeletal stress, damage to surrounding soft tissues, and misalignment. In particular, misalignment is a risk factor for noncontact injuries in taekwondo athletes because it causes the structural deformation of bones, abnormal arthrokinematics (the excessive motions between articulating joint surfaces in all three planes of motion), and improper force distribution in the lower extremity joints [10,11,12,13].

Although kicks are based on similar techniques and biomechanics, and a high incidence of injuries occurs in the lower extremities, epidemiological data in taekwondo have shown that injuries differ in terms of site and type depending on the subdiscipline [12,14]. In addition, previous studies have reported that navicular drop of the dominant leg, knee hyperextension, and excessive ROM of ankle dorsiflexion and plantarflexion are associated with lower extremity injuries in adolescent taekwondo sparring athletes [15,16]. These findings indicate that lower extremity alignment (LEA) influences the incidence of injuries in taekwondo due to variations in training regimes, athletic requirements, and kicking techniques among the subdisciplines.

While various studies have analyzed the biomechanics of kicks executed by sparring athletes to identify dynamic alignment and injury mechanisms, studies analyzing LEA according to subdisciplines are lacking [4,7,17]. Therefore, this study analyzed LEA in the dominant and nondominant legs of taekwondo athletes. This study hypothesized that differences in LEA exist among the dominant and nondominant legs of taekwondo athletes across the subdisciplines.

## 2. Materials and Methods

### 2.1. Participants

In total, 157 participants (63 sparring, 50 demonstration, and 44 poomsae athletes) were included in this study. The participants of this study were included if they were sparring, demonstration, or poomsae taekwondo athletes registered with the Korea Taekwondo Association in 2021 (Table 1). For power analysis, the required sample size was determined using a minimum statistical power of 0.8, α = 0.5, and a moderate effect size of 0.25. Thus, the required total sample size was 159 participants.

### 2.2. Procedure

Approval of the Institutional Review Board (IRB) was obtained prior to conducting this study (2-1040709-AB-N-01-202109-HR-072-06). The purpose of this study was explained to the participants prior to participation, and among the athletes (*n* = 185) who volunteered to participate, 157 were evaluated in sparring (*n* = 63), demonstration (*n* = 50), and poomsae (*n* = 44). Athletes who had lower extremity injuries were excluded (*n* = 28).

### 2.3. Measurement Tools

An athletic trainer with more than 10 years of field experience established an intraclass correlation coefficient (ICC) level for the measurement of LEA. The following tools were used to measure LEA characteristics: the software alignment, which uses a camera to assess the Q-angle, tibiofemoral angle, and genu recurvatum; a goniometer for tibial torsion, rearfoot, and forefoot; a bubble inclinometer for hip anteversion; a height gage for navicular drop; and a palpation meter (PALM) for pelvic tilt (Table 2).

### 2.4. LEA Measurement

The LEA measurement method used in this study has been previously described elsewhere [18]. The various LEA variables were measured in the standing, supine, and prone positions. To utilize the camera application, 8 mm colored stickers (red, blue, and green) were placed on the anatomical landmarks of each measurement. The participants were requested to wear spandex to facilitate these measurements. Both legs were measured and they were divided into dominant and nondominant legs. The leg that the athletes most comfortably and frequently used in kicking was defined as the dominant leg, whereas the contralateral leg was set as the nondominant leg (Table 3).

#### 2.4.1. LEA in the Standing Position

To measure the Q-angle, the midpoints of the patella, anterior superior iliac spine (ASIS), and tibia tuberosity were marked. The tibiofemoral angle was measured by marking the midpoints of the patella, between the ASIS and greater trochanter (GT), and between the medial and lateral malleolus. The rearfoot was measured using the midpoints of both malleoli, the calcaneus, and the lower third of the calf. The application detected the midpoint marks to measure each variable.

**Figure 1:**
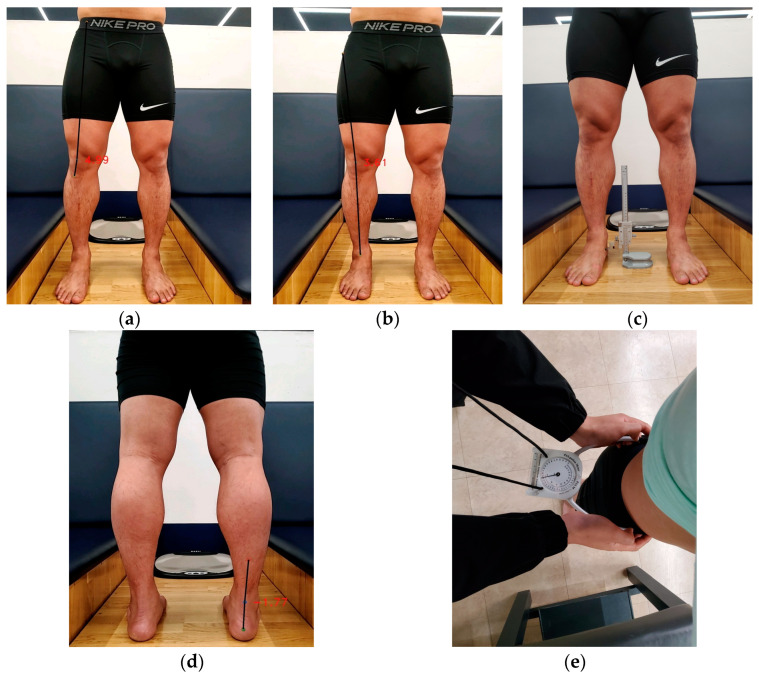
Measurement of lower extremity alignment in the standing position. (**a**) Q-angle. (**b**) Tibiofemoral angle. (**c**) Navicular drop test. (**d**) Rearfoot. (**e**) Pelvic tilt.

The navicular drop test calculated the difference between the height of the navicular tubercle when the subtalar joint was neutralized and the height measured when standing in a relaxed position. Pelvic tilt was measured as the angle between the ASIS and posterior superior iliac spine using PALM (Figure 1).

#### 2.4.2. LEA in the Supine Position

**Figure 2:**
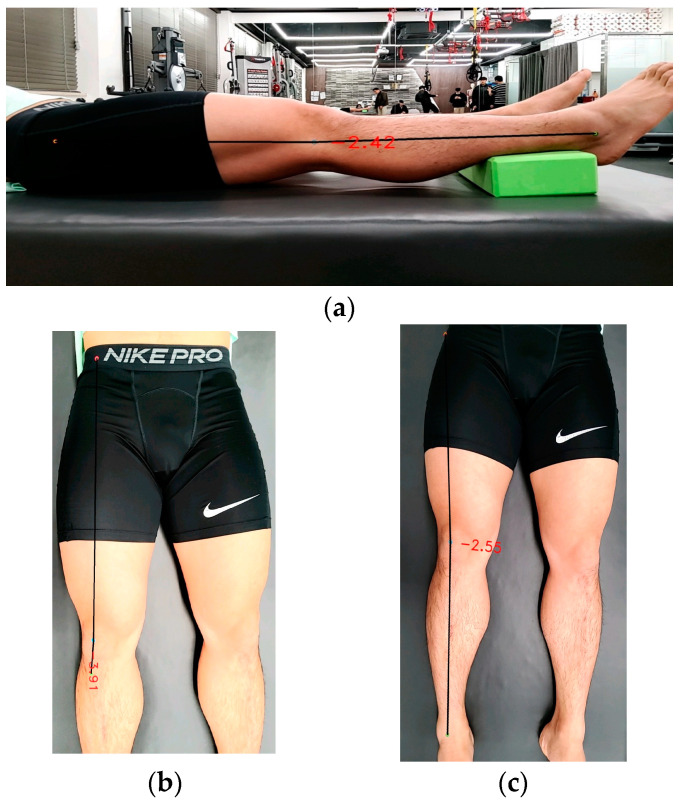
Lower extremity alignment measurement in the supine position. (**a**) Genu recurvatum. (**b**) Q-angle. (**c**) Tibiofemoral angle.

The genu recurvatum was measured by placing a foam roller under the ankle and marking the midpoint of the knee joint line, GT, and lateral malleolus (Figure 2). The application detected each mark to measure the variables.

#### 2.4.3. LEA in the Prone Position

**Figure 3:**
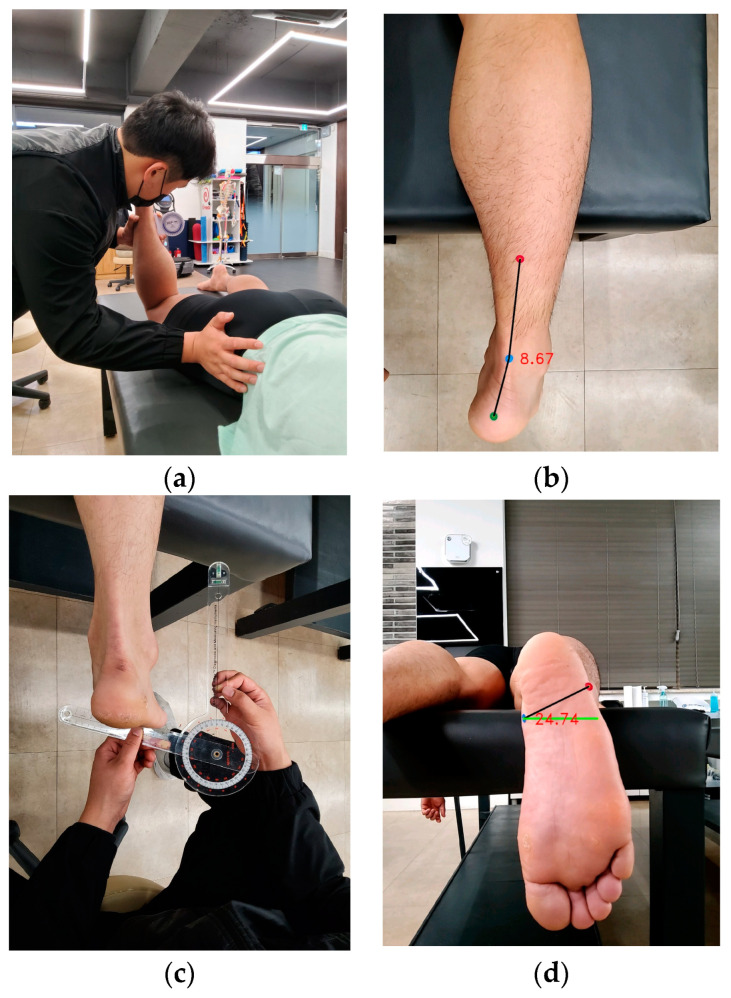
Lower extremity alignment measurement in the prone position. (**a**) Hip anteversion. (**b**) Rearfoot. (**c**) Forefoot. (**d**) Tibial torsion.

Hip anteversion was measured in the prone position. The femur was placed in a neutral position, the knee was flexed at 90°, and one hand was placed over the GT. The leg was rotated until the most predominant part of the GT was palpated. An inclinometer was then placed on the lower third of the medial surface of the tibia to measure the angle (Figure 3a). To measure the rearfoot, the femur and subtalar joint were placed in a neutral position. Then, dots were marked at 1/3 of the lower leg, the midpoints of both malleoli, and the calcaneus (Figure 3b). To measure the forefoot, the subtalar joint was placed in a neutral position, and the angle between the plantar surface of the forefoot and the imaginary axis was measured using a goniometer (Figure 3c). To measure tibial rotation, the femur was placed in a neutral position, and the angle formed by the imaginary line connecting both malleoli bones on the sole of the foot and the angle to the imaginary parallel line was measured (Figure 3d).

### 2.5. Statistical Analysis

Descriptive statistics (mean and standard deviation) were used to describe demographic characteristics. Separated one-way ANOVA was used to compare LEA among the taekwondo subdisciplines. For variables with a significant difference, the Scheffe post hoc test was performed to identify differences between each group.

## 3. Results

### Comparison of LEA among Taekwondo Athletes by Subdiscipline

In the dominant leg, the tibiofemoral angle in the standing (*F* = 4.38, *p* < 0.01) and supine (*F* = 5.89, *p* = 0.00) positions was greater in the poomsae group than in the sparring group (*p* < 0.05). Furthermore, the rearfoot (*F* = 5.62, *p* = 0.00) was higher in the sparring group than in the demonstration group (*p* < 0.05).

In the nondominant leg, the Q-angle in the supine position (*F* = 3.44, *p* < 0.05) was greater in the poomsae group than in the demonstration group. The tibiofemoral angle in the standing position (*F* = 10.17, *p* = 0.00) was greater in the poomsae group than in the sparring group. The tibiofemoral angle in the supine position (*F* = 10.13, *p* = 0.00) was greater in the poomsae group than the sparring and demonstration groups. The rearfoot in the standing position (*F* = 6.60, *p* = 0.00) was higher in the sparring group than in the demonstration group. Furthermore, the forefoot (*F* = 5.72, *p* = 0.00) was significantly higher in the demonstration group than in the poomsae group (Table 4).

## 4. Discussion

### 4.1. Comparison of LEA in Taekwondo Athletes’ Dominant Leg

In comparing the LEA of the dominant leg by specific taekwondo discipline, poomsae athletes showed a lower genu valgum alignment than sparring athletes, whereas sparring athletes showed a greater rearfoot varum alignment compared with demonstration athletes.

Poomsae has relatively static and predictable movements compared with sparring and demonstration, but is scored based on the height of the kick, which requires a wide ROM in the hip joint. Hip abductor muscle activation and strength influence the genu valgum and varum [19]. In other words, delayed onset and reduced strength in the hip abductor muscle will result in knee position alignment upon foot contact on the ground. In particular, the side kick is one of the most challenging kicking techniques in poomsae, with the medial rotation of the kicking leg resulting in an increased angular velocity of the femur and lower leg, followed by a decrease in the angular velocity at impact [20]. In addition, because impact must occur at the highest point of the kick, the genu valgum force is significant in the knee-straightening motion [21]. Previous studies have reported that the transferred momentum increases with the kick height, resulting in a large angular momentum, which is associated with muscle tension that represents the movement of the corresponding segment [20]. A dynamic genu valgum increases the valgus force, which is associated with an increased risk of anterior cruciate ligament (ACL) and medial collateral ligament (MCL) injuries [22,23]. Previous studies have reported that athletes who underwent ACL reconstruction exhibited a distinctly formed genu valgum, suggesting an increased risk of retear or rupture of the contralateral limb [22]. Kicking in poomsae likely exhibits a greater genu valgum alignment than that in sparring because the technique involves not only the height of the kick, but also the strength to maintain it, as judged by accuracy and expressiveness. This may also suggest that genu valgum alignment may be indicative of knee injury.

Sparring athletes showed a higher rearfoot varum alignment than demonstration athletes. The genu valgum is associated with the development of secondary injuries, such as foot malalignment and joint pathologies, such as instability [24,25]. Previous studies have shown that foot varum alignment assessed in an open kinetic chain indicates excessive pronation during the stance phase of running and that hip adduction is related to foot pronation [26]. The rearfoot varum is a risk factor for chronic ankle instability (CAI) and contributes to biomechanical changes, such as increased ankle inversion and increased lateral displacement of the center of pressure (COP) [27]. Another previous study reported that rearfoot misalignment causes compensatory action of the subtalar joint, which is involved in the normal function of the leg and foot during the gait cycle. Furthermore, excessive pronation of the subtalar joint inhibits foot supination and results in abnormal internal rotation of the tibia, which leads to patellar malalignment [28]. Among taekwondo athletes, sparring athletes perform kicking techniques using ground reaction force with the forefoot for the longest period of time, and forefoot pronation occurs rapidly and repeatedly, especially in situations such as maintaining distance, changing direction, and continuing skills. The forefoot and rearfoot are balanced in opposite positions, and the pronation of the forefoot, which occurs at the beginning of the stance phase of kicking, represents the movement of the rearfoot supination [29,30]. Thus, sparring athletes may develop a distinct rearfoot varum angle due to repetitive rotation of the foot on the ground from performing repeated continuous kicks. The rearfoot varum alignment may indicate injuries related to the lateral ankle, such as lateral ankle sprain and CAI.

### 4.2. Comparison of LEA in Taekwondo Athletes’ Nondominant Leg

The genu valgum angle of the nondominant leg was found to be statistically higher in poomsae athletes than in sparring and demonstration athletes. Poomsae is further subdivided into certified poomsae, which consists of basic taekwondo movements (kicking, striking, and blocking, etc.), and free poomsae, which combines poomsae and demonstration kicks to perform free techniques accompanied by music. In particular, certified poomsae is characterized by balanced attack and defense techniques in all four directions (front, back, and both sides), which enables attacks with the dominant and nondominant legs at the same frequency, unlike sparring and demonstration. However, the dominant leg in poomsae is more frequently used in kicking height and strength control, whereas excessive full extension and rotation occur in the supporting (pivot) leg with a high balance ability [25]. Techniques such as side kicks, front kicks, and Hakdariseogi with one-legged standing require higher strength and proprioception in the supporting leg [31]. A previous study confirmed that muscle fatigue in taekwondo athletes affects joint position sense and ROM in the flexion and extension of the knee joint [32]. Since the human body exhibits a crossed extension reflex chain response, in which the activation of the flexors on the ipsilateral side promotes the extensors on the contralateral side to maintain balance, one-legged skills in the poomsae cause valgus force by promoting extensors in the supporting leg [33]. From the perspective of functional anatomy, each segment on the ipsilateral side is characterized by opposite rotations to maintain balance. As the kick height increases, opposite rotations, such as external rotation in the ankle and internal rotation in the tibia, occur [21]. Previous studies have found that a higher kick results in a higher genu valgum angle in the supporting (pivot) leg, which affects hip–knee ROM and the simultaneous activity of the peripheral stabilizer muscles [21]. In addition, the supporting (pivot) leg undergoes excessive internal rotation of the tibia during kicking, and the time interval of the genu valgum moment is associated with a risk of ACL injury [20]. The nondominant leg of a poomsae athlete is more frequently used to maintain balance during high kick techniques and exhibits greater genu valgum alignment than that of sparring and demonstration athletes. This may be related to the incidence of knee injuries such as ACL and MCL.

In this study, we found that sparring athletes have greater rearfoot varum alignment than demonstration athletes. These results are similar to those of previous studies that found no difference in kinematic variables between the dominant and nondominant legs of sparring athletes [31,32]. The kicks of sparring athletes combine throw-like and push-like motions, with a high frequency of performing such techniques from an undefined height and distance [32]. Previous reports have shown that the dominant leg exhibits a high reaction speed and impact force [34], but the frequency of attacking with the nondominant leg is increasing because of anomalous kicks (monkey kick, raised kick, and scorpion kick, etc.) performed after the recent introduction of electronic protectors. This phenomenon may be related to the rearfoot varum alignment of both the dominant and nondominant legs in sparring athletes.

In this study, we confirmed that demonstration athletes showed a greater forefoot varum alignment than poomsae athletes. Breaking, which is considered to be the core performance in demonstration, represents a high level of skills, including spinning and connecting kicks in the air and on the ground. In particular, kicking in the air involves approaching followed by a stable landing, and the more time spent in the air, the better the expression of the kicks [33]. Demonstration kicking is characterized by jumping with the attacking leg, followed by a strike and landing, unlike poomsae and sparring. A single-footed landing results in decreased dorsiflexion as the COP line moves forward [34]. A sustained decrease in dorsiflexion contributes to dynamic postural control deficits and can lead to CAI [35]. A kinematic analysis of the demonstration kick revealed that reduced ROM in ankle dorsiflexion with weight bearing is associated with reduced knee flexion during jumping and landing [34]. Reduced dorsiflexion ROM and forefoot varum misalignment are associated with an increased risk of ankle impingement syndrome and metatarsal stress fractures [36]. Forefoot supination of the weight-bearing leg during jumping and landing causes damage and injury to the lateral ankle soft tissues and is associated with the development of pathologies such as lateral ankle sprain, CAI, and ankle impingement syndrome [37,38].

The current study has some limitations. First, the sex ratio in each subdiscipline was not matched; therefore, the differences in LEA by sex could not be determined. Since the human body has different structural characteristics between men and women, a sex-matched analysis may reveal another dimension of meaningful results that would facilitate coaches and clinicians’ understanding of the structural differences between sexes. Second, the specialized kicks in each subdiscipline were excluded in the analysis. Analyzing the specialized kicks in future studies may clarify the relationship between kick and alignment, because each specialized kick has differences in speed, power, attacking, and so on. Third, a history of surgery or injury to the lower extremity was excluded from the data. To predict injury from a history of surgery or injury, regular monitoring of LEA is recommended, so that the LEA factors that increase the risk for injury can be identified within a taekwondo team.

## 5. Conclusions

In summary, differences were found in the LEA characteristics among taekwondo athletes of all subdisciplines. The results of this study indicate the necessity for coaches and clinicians to identify the LEA characteristics of each taekwondo subdiscipline to design tailored training programs that would address specific areas.

## Figures and Tables

**Table 1:** Demographic characteristics of taekwondo athletes.

Variables	Sparring			Demonstration			Poomsae		
	Male	Female	Total	Male	Female	Total	Male	Female	Total
*n* (%)	52 (82.5)	11 (17.5)	63 (100)	43 (86.0)	7 (14.0)	50 (100)	27 (61.4)	17 (38.6)	44 (100)
Age (years)	21.25 ± 1.23	21.45 ± 1.29	21.29 ± 1.24	21.37 ± 0.98	20.71 ± 0.95	21.24 ± 1.00	21.26 ± 0.94	21.59 ± 1.12	21.39 ± 1.02
Height (cm)	179.91 ± 5.53	167.27 ± 5.96	177.7 ± 7.37	173.90 ± 5.43	161.13 ± 3.63	172.1 ± 6.85	172.87 ± 5.34	161.41 ± 4.78	168.44 ± 7.59
Weight (kg)	73.45 ± 10.54	57.84 ± 8.97	70.72 ± 11.84	68.33 ± 6.88	55.09 ± 5.71	66.48 ± 8.13	68.57 ± 10.94	57.14 ± 8.14	64.15 ± 11.35
BMI (kg/m^2^)	22.68 ± 2.96	20.59 ± 2.37	22.31 ± 2.96	22.58 ± 1.92	21.2 ± 1.71	22.38 ± 1.93	23.08 ± 4.18	21.88 ± 2.35	22.62 ± 3.60
Body fat (%)	16.91 ± 6.72	24.25 ± 8.06	18.19 ± 7.45	16.25 ± 5.95	27.24 ± 5.32	17.79 ± 6.98	18.23 ± 7.60	30.49 ± 4.65	22.97 ± 8.90

Data are expressed as mean ± standard deviation. BMI, body mass index.

**Table 2:** LEA measurement tools.

Tool	Manufacturer	Variables
Application	Alignment, alpha version 1.0.2, Yonsei University, Seoul, Republic of Korea	Q-angle, genu recurvatum, tibiofemoral angle, rearfoot
Goniometer	Baseline Evaluation Instruments, White Plains, NY, USA	Tibial torsion, rearfoot, and forefoot
Bubble Inclinometer	Baseline Evaluation Instruments, White Plains, NY, USA	Hip anteversion
Height gage	H4-20, Mitutoyo Mfg. Co., Ltd., Tokyo, Japan	Navicular drop test
Palpation meter	Baseline Evaluation Instruments, White Plains, NY, USA	Pelvic tilt

**Table 3:** Intraclass correlation coefficient for LEA measurements.

Variables	Position	ICC (2,1)
Hip anteversion	Prone	0.97
Q-angle	Standing	0.94
	Supine	0.87
Tibiofemoral angle	Standing	0.90
	Supine	0.96
Genu recurvatum	Supine	0.89
Tibial torsion	Prone	0.90
Navicular drop test	Standing	0.98
Rear foot	Standing	0.85
	Prone	0.91
Forefoot	Prone	0.87
Pelvic tilt	Standing	0.97

**Table 4:** Comparison of LEA among taekwondo athletes.

Variables	Group	Dominant Leg				Nondominant Leg			
		Mean (SD)	*F*	*p*	Post Hoc	Mean (SD)	*F*	*p*	Post Hoc
Hip anteversion	SG	9.19 (4.19)	0.64	0.53	No Sig.	9.08 (5.08)	2.03	0.14	No Sig.
	DG	9.86 (4.46)				8.32 (4.17)			
	PG	10.16 (5.18)				10.27 (4.76)			
Q-angle (ST)	SG	17.76 (6.86)	1.92	0.15	No Sig.	17.29 (6.00)	2.82	0.06	No Sig.
	DG	19.18 (7.28)				16.90 (7.72)			
	PG	20.51 (7.54)				20.08 (7.68)			
Q-angle (SU)	SG	17.46 (6.20)	2.62	0.08	No Sig.	15.99 (4.82)	3.44	0.04	DG < PG
	DG	18.93 (6.00)				14.82 (5.67)			
	PG	20.24 (6.53)				17.89 (6.83)			
TFA (ST)	SG	6.84 (3.12)	4.38	0.01	SG < PG	6.14 (2.90)	10.17	0.00	SG < PG
	DG	7.67 (2.51)				7.37 (2.52)			
	PG	8.54 (3.08)				8.68 (3.13)			
TFA (SU)	SG	6.28 (2.59)	5.89	0.00	SG < PG	5.67 (2.46)	10.13	0.00	SG, DG < PG
	DG	6.80 (2.78)				6.22 (2.71)			
	PG	8.12 (2.92)				8.04 (3.13)			
GR	SG	1.49 (3.44)	2.60	0.08	No Sig.	0.37 (3.35)	2.28	0.11	No Sig.
	DG	1.59 (3.63)				0.43 (3.42)			
	PG	−0.07 (4.94)				−1.01 (4.49)			
ND	SG	0.73 (0.36)	0.10	0.90	No Sig.	0.75 (0.47)	2.58	0.08	No Sig.
	DG	0.70 (0.38)				0.66 (0.40)			
	PG	0.76 (0.94)				0.56 (0.35)			
Rearfoot (ST)	SG	1.72 (2.57)	5.62	0.00	DG < SG	1.72 (2.77)	6.60	0.00	DG < SG
	DG	−0.05 (3.43)				−0.16 (2.97)			
	PG	0.96 (2.21)				0.77 (2.42)			
Rearfoot (PR)	SG	4.33 (3.15)	3.15	0.05	No Sig.	4.14 (2.85)	2.33	0.10	No Sig.
	DG	3.19 (2.67)				3.07 (4.57)			
	PG	3.18 (2.36)				8.50 (4.07)			
Forefoot	SG	10.94 (4.77)	0.41	0.66	No Sig.	10.28 (4.68)	5.72	0.00	PG < DG
	DG	11.36 (5.01)				11.63 (4.57)			
	PG	10.47 (4.33)				8.50 (4.07)			
Pelvic tilt	SG	7.20 (3.03)	1.13	0.33	No Sig.	6.93 (3.03)	0.22	0.80	No Sig.
	DG	7.83 (4.14)				7.38 (4.58)			
	PG	6.77 (3.10)				7.02 (3.26)			
Tibia torsion	SG	19.16 (7.40)	1.41	0.25	No Sig.	17.90 (6.90)	0.79	0.45	No Sig.
	DG	20.46 (7.21)				16.56 (6.47)			
	PG	17.95 (6.92)				16.29 (8.39)			

Abbreviations: SD, standard deviation; TFA, tibiofemoral angle; GR, genu recurvatum; ND, navicular drop; ST, standing; PR, prone; SU, supine; SG, sparring group; DG, demonstration Group; PG, poomsae Group; and No Sig., no significant difference.

## Data Availability

Data is unavailable due to privacy or ethical restrictions.
